# Special Issue: Fungal Pathogenesis in Humans: The Growing Threat

**DOI:** 10.3390/genes10020136

**Published:** 2019-02-12

**Authors:** Fernando Leal

**Affiliations:** Instituto de Biología Funcional y Genómica/Dpto. Microbiología y Genética, (CSIC/USAL), Zacarías González 2, 37007 Salamanca, Spain; fleal@usal.es

Approximately 150 fungal species are considered as primary pathogens of humans and animals. The variety of infections that they may cause ranges from localized cutaneous, subcutaneous or mucosal infections to systemic and potentially fatal diseases. Many fungi are also able to cause lesions when abnormal patient susceptibility exists or after traumatic colonization of the fungus (for a comprehensive review on Medical Mycology, see Kwon-Chung and Bennet, [1]). Fungi that infect immunocompromised patients are referred to as opportunistic pathogens. The number of opportunistic fungi has recently increased due to the arrival of new and growing populations of immunocompromised hosts. In this special issue, we have attempted to compile a collection of new studies investigating the role of some virulence traits and their molecular mechanisms of action in the pathogenic outcome of fungal infections.

The term candidiasis refers to a wide clinical spectrum of infections that can be acute or chronic, superficial (cutaneous, oropharyngeal, vulvovaginal, ocular) or deep (esophageal, gastrointestinal, respiratory, urinary, etc.) and can affect either normal or immunosuppressed individuals. The major etiologic agent is *Candida albicans*, which is part of the normal human mycobiota. However, several other species are frequently encountered in certain clinical diseases (*Candida parapsilosis*, *Candida glabrata*, *Candida tropicalis*, *Candida lusitaniae*). Here, three different aspects of *Candida* infections are examined: the maintenance of chromosomal integrity; biofilm formation as a form of survival; and the establishment of new models of infection as an alternative to mice. Ciudad et al. [2] address the problem of repairing the alkylation base damage in the genome of *C. albicans*. After analyzing the response of three homologous recombination (HR) mutants to chromosomal damage caused by methyl methanesulfonate (MMS), these authors propose that repair takes place through a mechanism (possibly base excision repair) that does not involve homologous recombination. Biofilm formation allows *Candida* to adhere to and proliferate on medical devices and host tissues. Biofilms are constituted of a mixture of filamentous and yeast cells that surround themselves with an extracellular matrix, which provides a remarkable degree of resistance to antifungal drugs. Rodrigues et al. [3] evaluate the role of *C. parapsilosis* genes associated with the production of the biofilm matrix by monitoring their expression levels in response to treatment with antifungal drugs. They concluded that although beta-1,6-glucans and mannans are an essential part of both cells and the biofilm matrix, β-1,3-glucan seems to play a more important role in biofilm resistance to antifungal drugs. Chong et al. [4] provide a detailed review of numerous studies on *C. albicans* biofilms, which includes the analysis of the transcriptome, whole genome sequencing, functional genomic approaches to identify critical regulatory genes and comparative genomics analysis. In addition, recently discovered pathways and genes involved in the pathogenesis of the fungus are described and future directions in the development of therapeutics are suggested. Finally, Souza et al. [5] confirm the suitability of *Caenorhabditis elegans* as an alternative to the use of mouse models in pathogenesis studies, which can be infected and killed by three species of the *C. parapsilosis* complex. The progression of the infection was determined by histological examination and the immune response of *C. elegans* was monitored by analyzing gene expression. Early treatment with antifungal drugs was also found to be effective in this model.

Aspergilli are ubiquitous fungi found within our environment, which humans are continuously being exposed to. However, the diseases caused by these fungi are relatively uncommon and the severe, invasive form of these diseases is almost always confined to immunosuppressed individuals. There are three main manifestations of disease: an allergic response to inhaled aspergilli; the colonization of air spaces within the body; and tissue invasion by the fungus. *Aspergillus fumigatus* represents the most frequent etiologic agent of both noninvasive and invasive aspergillosis while *Aspergillus flavus* and *Aspergillus niger* can also provoke invasive pulmonary aspergillosis in immunosuppressed patients. Classical genetic studies on the genera soon progressed to the molecular level, which allowed for the discovery of several mechanisms involved in virulence and pathogenesis pathways. In this special issue, a review and an original article are included, which emphasize the importance of whole genome comparative studies for identifying pathogenic properties based on differences in DNA sequences. García-Rubio et al. [6] perform Whole Genome Sequencing (WGS) on more than a hundred *A. fumigatus* strains and highlight the importance of choosing the most suitable reference genome for analyzing the genetic differences between *A. fumigatus* strains, their genetic background and the development of antifungals resistance. Furthermore, Ashu and Xu [7] propose in their concept paper that these types of studies could be expanded to devise molecular epidemiology and experimental evolution methods that are useful for managing the *Aspergillus* threat. These authors provide a framework for such a purpose that implies the development of rapid and accurate diagnostic tools to genotype the infectious pathogen to the level of the species and the individual as well as drug susceptibility patterns. One of the recently described virulence mechanisms relies on the ability of *Aspergillus* to obtain essential ions (mainly Fe and Zn) from the extremely limited supply of micronutrients existing in host tissues. Vicentefranqueira et al. [8] determine how the ZafA transcription factor of *A. fumigatus* regulates zinc homeostasis and its importance for virulence. The combined use of microarrays, Electrophoretic Mobility Shift Assays (EMSA), DNAse I footprinting assays and in silico tools have been essential for obtaining a better understanding of the regulation of the homeostatic and adaptive response of this fungus to zinc starvation.

Cryptococcosis is the fourth most commonly recognized cause of life-threatening infections among AIDS patients and different types of immunosuppression are the predisposing factors influencing the rate of infection in non-AIDS patients. Cryptococcosis are infections caused by the encapsulated fungus *Cryptococcus neoformans* that occur after the spores are inhaled into the lungs. This causes pneumonia and frequently spreads hematogenously to the brain and meninges, causing meningoencephalitis. The role of small RNAs and the mechanisms by which some reach their target are addressed in two papers included within this issue. Using next-generation sequencing and bioinformatics tools, Huo et al. [9] report the existence of stable circRNAs in the genome of *Cryptococcus neoformans* for the first time. These RNAs were hosted in genes that were mainly responsible for primary metabolism and ribosomal protein production. Highly transcribed circRNAs from GTPase and RNA debranching enzyme genes were discovered. The role of these small RNAs in pathogenesis remains open for discussion. Extracellular vesicles (EVs) have been found to play important roles in crosstalk between different types of cells and tissues from the same or even different species. Many fungi use these vesicles as carriers for polysaccharides, proteins and RNAs, but their implication in pathogenesis is still not clear. Peres da Silva et al. [10] investigate if EV-mediated RNA export in *C. neoformans* was functionally connected with the Golgi reassembly and stacking protein (GRASP). The results obtained after analyzing the mutants that have defective GRASP synthesis and autophagic mechanisms suggest that GRASP, but not the autophagy regulator, is involved in the EV-mediated export of RNA. This function as a key regulator of unconventional secretion in eukaryotic cells is a new finding.

Other fungal infections that are not as widespread or as fatal as those caused by *Candida, Aspergillus* and *Cryptococcus* are also mentioned in this special issue. Mucormycosis are a set of infections caused by members of the order Mucorales in patients with serious underlying conditions. Vascular invasion by hyphae results in infarction and necrosis of tissues. Although *Rhizopus oryzae* is the most common causal agent of human mucormycosis, *Mucor circinelloides* isolates have been associated with outbreaks of the disease. Unfortunately, the genetic manipulation of these basal fungi is not well established, which has impeded the study of their virulence traits and pathogenesis mechanisms. Partially overcoming these challenges, Binder et al. [11] generate and functionally characterize a bioluminescent strain of *M. circinelloides* designed to be used in the monitoring of real-time and non-invasive infection in insect and murine models and in the testing of antifungal drug efficacy. Dermatophytes are fungi capable of infecting keratinized tissues, such as the epidermis, hair and nails, without affecting subcutaneous or deep tissues, whereas *Trichophyton rubrum*, the major etiologic agent of human ringworm, causes chronic lifetime infections. It is also worth mentioning the work by Petrucelli et al. [12] who describe a *T. rubrum*- HaCat keratinocyte co-culture, which is used to mimic the natural fungal–host interaction, where dual RNA-seq technology was used to evaluate the transcriptomes of both organisms. These authors found that some keratinolytic proteases and glyoxylate cycle encoding genes that may improve nutrient assimilation and fungal survival and colonization were induced in the fungus. In human keratinocytes, some genes involved in the epithelial barrier integrity were inhibited, whereas others that played a role in antimicrobial activity were induced.

A problem that is common to all fungal infections is their resistance to antifungals, with growing concern focused on how to treat most of the aforementioned diseases. Multidrug resistance transporters (MDRs) are key elements in mediating fungal resistance to pathogenesis-related stresses, a topic that was well described by Cavalheiro et al. [13]. These authors emphasize the importance of these transporters beyond the role of drug resistance and summarize their relevance in pathogenesis traits, such as resistance to host niche environments, biofilm formation, immune evasion and virulence.

I would like to express my deep appreciation for all of the hard work carried out by the investigators included in this special issue and those who were not due to different circumstances.

I hope that their enthusiasm and dedication to fungal research will encourage many young mycologists to apply the different approaches mentioned herein to the fascinating field of human fungal pathogenesis.
